# The immune evasion roles of *Staphylococcus aureus* protein A and impact on vaccine development

**DOI:** 10.3389/fcimb.2023.1242702

**Published:** 2023-09-27

**Authors:** Alex Bear, Thomas Locke, Sarah Rowland-Jones, Simone Pecetta, Fabio Bagnoli, Thomas C. Darton

**Affiliations:** ^1^ Department of Infection, Immunity and Cardiovascular Disease, The University of Sheffield, Sheffield, United Kingdom; ^2^ GlaxoSmithKline (GSK) Srl, Siena, Italy

**Keywords:** *Staphylococcus aureus*, Protein A, immune evasion, super antigen, vaccine development, B cells

## Abstract

While *Staphylococcus aureus* (*S. aureus*) bacteria are part of the human commensal flora, opportunistic invasion following breach of the epithelial layers can lead to a wide array of infection syndromes at both local and distant sites. Despite ubiquitous exposure from early infancy, the life-long risk of opportunistic infection is facilitated by a broad repertoire of *S. aureus* virulence proteins. These proteins play a key role in inhibiting development of a long-term protective immune response by mechanisms ranging from dysregulation of the complement cascade to the disruption of leukocyte migration. In this review we describe the recent progress made in dissecting *S. aureus* immune evasion, focusing on the role of the superantigen, staphylococcal protein A (SpA). Evasion of the normal human immune response drives the ability of *S. aureus* to cause infection, often recurrently, and is also thought to be a major hindrance in the development of effective vaccination strategies. Understanding the role of *S. aureus* virulence protein and determining methods overcoming or subverting these mechanisms could lead to much-needed breakthroughs in vaccine and monoclonal antibody development.

## 1 Introduction

**Figure 1:**
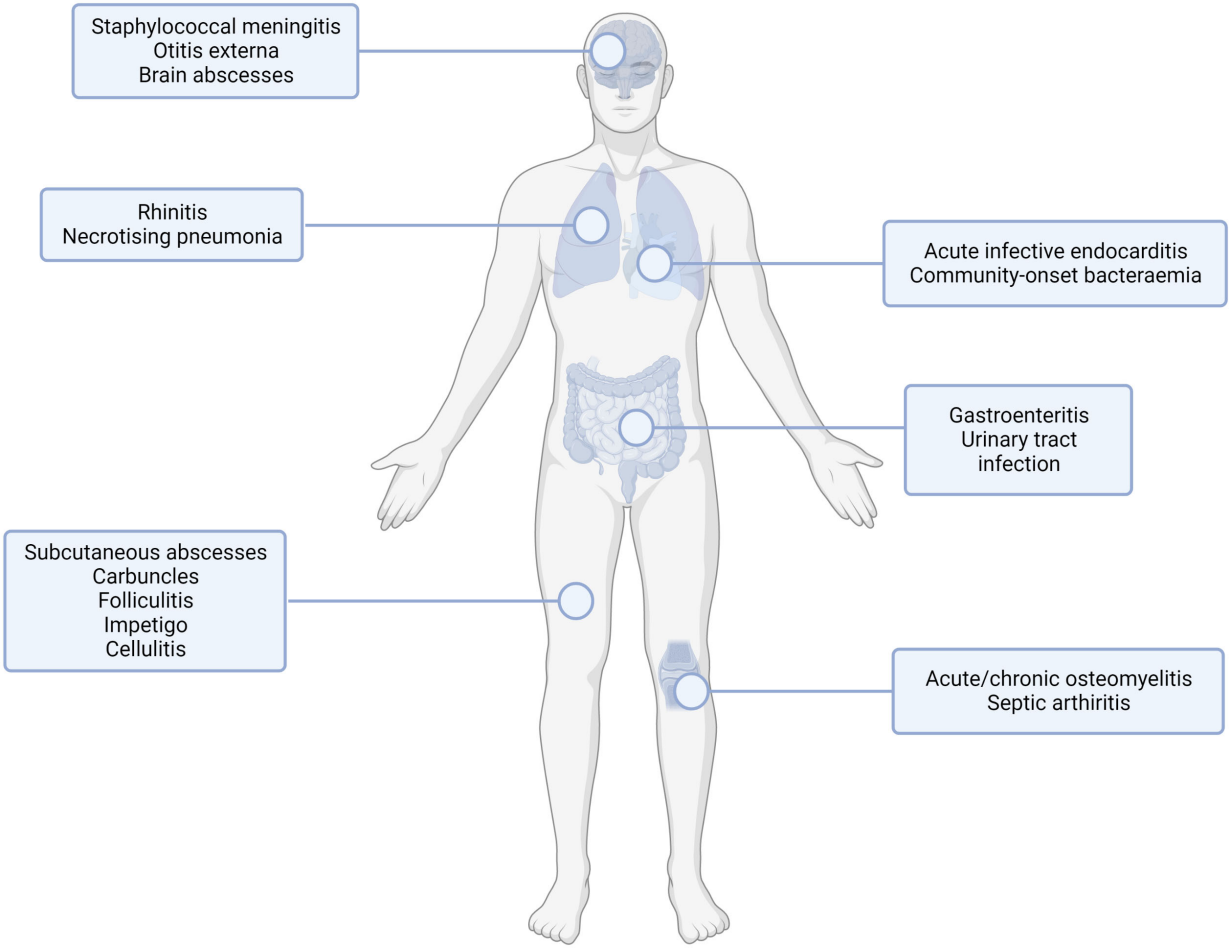
Common sites of staphylococcal infection. *S. aureus* infection can lead to multiple infection endpoints depending on the route of entry and the tissue infected. This figure shows the type of infection (infections of the internal organs, skin infections, orthopaedic infections) with examples for the subset of infection type shown. Created with BioRender.com.

*Staphylococcus aureus* is commonly found in the commensal flora of the human skin, nasopharynx, and gastrointestinal tract (Raineri et al., 2022). Occasionally, following breakdown of the epithelial barrier or inoculation through it, opportunistic infection may occur, resulting in one or more syndromes, as shown in **Figure 1**. *S. aureus* is a major opportunistic pathogen of humans, causing clinical manifestations from common skin infections such as impetigo or cellulitis to more severe and frequently life-threatening conditions such as infective endocarditis and sepsis. Recent analysis has demonstrated that *S. aureus* is the leading bacterial cause of death in 135 developing and developed countries worldwide (Collaborators GBDAR, 2022). A high burden of *S. aureus* infection is still associated with healthcare exposure including elective and emergency surgical treatment (Dreyfus et al., 2021).

Despite the introduction of evidence-based prevention measures, including bundles of care to prevent infection in hospitalised patients (Friedrich, 2019; Locke et al., 2022), rates of invasive *S. aureus* infection due to methicillin-susceptible *S. aureus* (MSSA) are continuing to increase year on year in the UK. While the incidence of MSSA and methicillin-resistant *S.* aureus (MRSA) bloodstream infection was diminished by the COVID-19 pandemic, UK rates have now returned to pre-pandemic levels reaching 12,956 reported cases in 2021/2022, more than 30% higher than 10 years ago (UK Health Security Agency, 2022). These national data are only a partial representation of *S. aureus* burden however, as they do not include non-bloodstream infections such as skin and soft tissue infection (SSTI). Most types of *S. aureus-*associated SSTI are community-acquired and include abscesses, impetigo, boils, cellulitis or folliculitis (Jauneikaite et al., 2020). True numbers of cases are difficult to ascertain as minor infections are frequently treated empirically (without diagnostic samples being collected), leaving the full burden of *S. aureus* infection unknown.

The development of an effective vaccine to prevent *S. aureus* infection would be an invaluable tool for reducing the burden of infection and associated healthcare costs and antimicrobial consumption. A key issue in developing an effective vaccine lies in the multiple virulence factors that *S. aureus* can leverage to evade the immune system (Anderson et al., 2012). Staphylococcal protein A (SpA) is an important component in immune evasion with myriad effects including acting as a B cell superantigen. Superantigens are a large and varied group of proteins that all exhibit the ability to strongly bind and activate the immune system in a non-specific manner. *S. aureus* production of superantigens prevents the human immune response’s ability to correctly identify and counter other *S. aureus* antigens. SpA protein, through the role it plays in impairing effective antibody generation and subverting a fully functional B-cell response against *S. aureus*, has therefore become of great interest in understanding how the human immune response to *S. aureus* evolve.

This review will explore the role that *S. aureus* proteins play in the evading the human immune system such that infection does not lead to protective immunity. We will focus on the immune evasion factor staphylococcal protein A (SpA) and explore the implications for successful vaccine development strategies.

## 2 Humans have evolved protective immune responses to counter infection by bacteria

**Figure 2:**
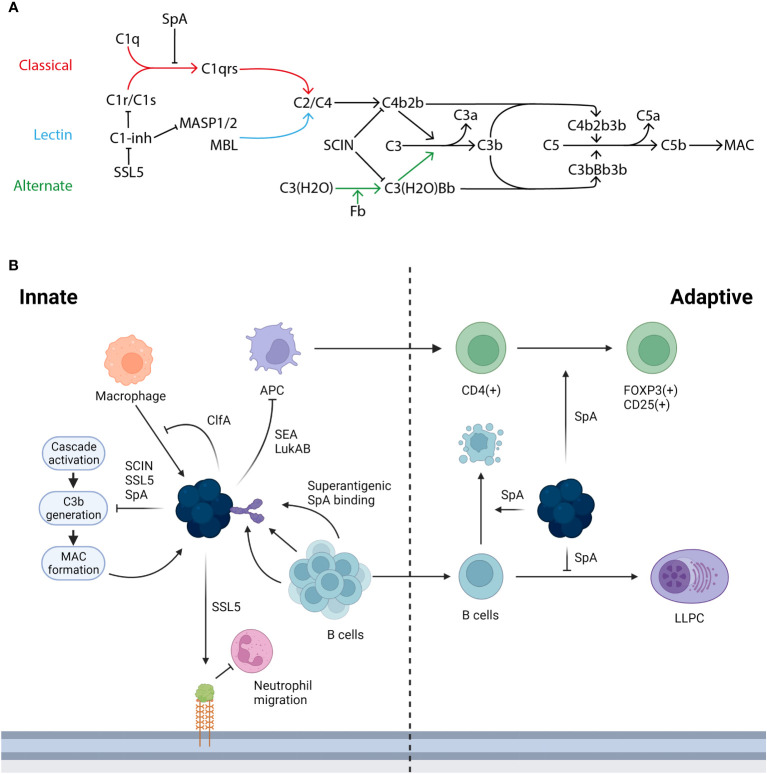
The immune response to *S. aureus* infection and the ways in which its proteins interact. **(A)** There are three activation pathways for the complement cascade; the classical (marked in red), the lectin (marked in light blue), and the alternative (marked in green). The classical pathway is activated when C1q binds the IgG generated by infection and forms the C1 complex with C1r/C1s, which goes on to convert C2/C4 to C4b2b. This process can be inhibited by SpA stopping the formation of the C1 complex with an antibody and also by C1-inhibitor (C1-inh), which is in turn blocked by staphylococcal superantigen-like protein 5 (SSL5). In the lectin pathway mannan-binding lectin serine protease 1/2 (MASP1/2) or mannose-binding lectin (MBL) acts as the initiator and converts C2/C4 to C4b2b. In the alternative pathway continuous low level activation leads to the conversion of C3(H2O) to C3(H20)Bb. Both C4b2b and C3(H2O)Bb then go on to convert C3 to C3a and C3b: this conversion can be blocked by staphylococcal complement inhibitor (SCIN). C3b then complexes with the two C3 convertases to form C5 convertases with converts C5 to C5a and C5b. C5b goes on to form the membrane attack complex (MAC). Adapted from Shinjyo, Kagaya and Pekna, 2021 (Shinjyo et al., 2021). **(B)** *S. aureus* is able to interact and interfere with both the innate and adaptive immune response in a number of ways. During the innate response macrophages (marked in orange) can target *S. aureus*, this in turn is blocked in its action by ClfA, additionally the initial innate response can be hampered by the action of SSL5 degrading PSGL-1 reducing the ability of neutrophils (marked in pink) to migrate into the infected tissue. A key part of the immune response is through the generation of B cells (marked in light blue) and the subsequent generation of antibodies however this process is hampered by the superantigenic binding by SpA, the B cell mediated immune response is further inhibited by SpA due to the role it plays in generating a clonal expansion and collapse. SpA plays a further role in limiting the adaptive immune response, by altering and downregulating the factors required in the niche for an LLPC to form. Further *S. aureus* proteins such as SEA and LukAB inhibit the transition from an innate to adaptive response targeting APC (marked in purple) death with further dysregulation of the adaptive T cell response mediated by SpA.

Bacteria such as *Streptococcus pneumoniae* or *Haemophilus influenzae* activate multiple components of the human immune response. Firstly, the innate immune system has the capacity to identify the bacteria as a foreign body; a key receptor for this process is the toll-like receptor 2 (TLR2), expressed on a wide variety of immune cells including monocytes and macrophages in addition to non-immune cells such as keratinocytes. TLR2 recognises pathogen associated molecular patterns (PAMPS) on the bacterial surface (Yoshimura et al., 1999). TLR2 is flexible in the PAMPS it can recognise due to its ability to form heterodimers with other TLRs and non-TLR molecules (Oliveira-Nascimento et al., 2012). The pathogen is then phagocytosed and lysed, resulting in inflammation and the release of cytokines. This process of PAMP recognition, alongside others, can trigger the activation of the complement cascade, which plays a multitude of roles in combatting infection. The complement system has 3 major pathways of activation, the classical pathway, the alternative pathway, and the lectin pathway, with the classical being associated with adaptive immunity and the alternative and lectin being activated in the absence of antibody during the innate response. Each of these pathways results in the conversion of C3 to C3a and C3b, which go on to have further downstream effects, as shown in **Figure 2**. The complement system is essential for an effective immune response and many bacteria leverage it to aid in their evasion of the immune response (Rosbjerg et al., 2017).

The immune response then begins to transition to an adaptive one, with the subsequent generation of a specific immune response primarily through the presentation of antigens in lymph nodes by dendritic cells. The adaptive immune response is largely mediated by B and T cells to generate antibodies via plasma B cells, manage the inflammatory response through neutrophils and macrophages, destroy the bacteria with natural killer cells, and maintain long lasting immune memory utilising long-lived plasma cells and memory T- and B-cells.

This coordinated and wide-ranging ability to respond to new pathogens results in an effective response: however, bacteria, in common with other pathogens, have evolved to counter the immune system’s ability to perform effectively. A key area exploited by bacteria is the evasion of neutrophils and the management of chemotaxis. An array of different evasion mechanisms exist which include, for example, the prevention of initial neutrophil recruitment by degradation of P-selectin glycoprotein ligand-1 (PSGL-1) by *Streptococcus pneumoniae* zinc metalloproteinase, ZpmC (Surewaard et al., 2013), or the suppression of key cytokines such as gamma interferon by the virulence factor designated suppressive factor 1 of *Actinobacillus actinomycetemcomitans* (Kurita-Ochiai and Ochiai, 1996).

## 3 *S. aureus* has evolved a number of mechanisms to evade the immune system

*S. aureus* is particularly suited to evade the immune system, as it possesses a suite of proteins that can subvert both innate and adaptive responses (**Table 1**). For example, staphylococcal complement inhibitor (SCIN) plays a key role in undermining key C3 convertase enzymes, as shown in **Figure 2**, preventing important stages such as C3b deposition and C5a generation (Jongerius et al., 2009). Other staphylococcal proteins such as staphylococcal superantigen-like protein 5 (SSL5) disrupt other aspects of the innate response; similarly to *S. pneumoniae* ZpmC, SSL5 is able to bind PSGL-1 although without leading to degradation (Bestebroer et al., 2007).

**Table 1:** *S. aureus* immune evasion proteins.

*Protein or protein family*	*Abbreviation*	Protein target
Extracellular adhesion protein	Eap	Leukocyte migration and early immune response
Staphylococcal superantigen-like proteins	SSL	Variety of targets primarily in the complement cascade and innate response
Staphylococcal complement inhibitor	SCIN	Complement cascade
Clumping factor A	ClfA	Primarily fibrinogen with some effect on complement regulator I

This summarises the proteins discussed in this manuscript with the name of either the protein or protein family in the first column, the abbreviation used to discuss it in the second column, and the general target of the protein in the context of immune evasion.

The key transition from an innate to an adaptive immune response is also targeted by *S. aureus* evasion mechanisms. These include the disruption of dendritic cell antigen presentation by virulence factors such as staphylococcal enterotoxin A-depleting Langerhans cells (Pickard et al., 1994), and the ability of leukocidin A/B in targeting cell membranes resulting in dendritic cell death (Dumont et al., 2011). Immune system evasion both enables the initial infection to become established and ensures that subsequent adaptive responses and long-lasting immunity established by B and T cells are disrupted.

Some examples of *S. aureus* vaccine development have focused on these proteins. In particular, the NDV-3 vaccine candidate by NovaDigm therapeutics is designed to replicate Als3p of *Candida albicans*, which shares structural and sequence homology with clumping factor A (ClfA). ClfA is an adhesin involved in fibrinogen binding. Furthermore, it binds to complement control protein, factor I. By doing so ClfA increases cleavage of C3b deposited on *S. aureus* cells dampening its phagocytosis. NDV-3 has primarily been proposed for use in protection against SSTI’s caused by *S. aureus* and has so far been found to be safe and immunogenic (Schmidt et al., 2012). Clinical trials have also demonstrated that this vaccine provides robust protection against vulvovaginal candidiasis and ongoing trials are examining its potential to prevent nasal acquisition of *S. aureus* and thus break the chain of carriage transmission (Yeaman et al., 2014). A key barrier to the development of effective vaccines is the ability of *S. aureus* to produce numerous immune evasion proteins, including SpA.

## 4 SpA is able to manipulate the immune response to favour infection

SpA is a *S. aureus* cell wall protein originally isolated by Verwey in 1940 (Verwey, 1940), following chemical extraction and precipitation from a pathogenic ‘type A’ strain. Hitherto, differences in pathogenicity were thought to be related to carbohydrate production, but Verwey demonstrated the production of a protein able to non-specifically bind antibody. Subsequent comparison with staphylococcal antigens isolated by Jensen in 1959 (Jensen, 1958), demonstrated similarity with antigen A (after which SpA is named) and confirmed the substance as a protein located on the cell wall of certain *S. aureus* bacterial strains (Löfkvist and Sjöquist, 1962). Early studies demonstrated the immune evasion potential of SpA, including ability to inhibit opsonophagocytosis *in vitro* (Dossett et al., 1969).

### 4.1 SpA structure and function

SpA typically exists as a highly conserved cell wall protein but can also be secreted in an extracellular form where it can have an important role as a superantigen. Given in an unaltered and purified form, SpA is toxic to human and animals inducing an anaphylactic response due to the widespread cross-binding of anti-V_H_3 idiotype antibodies (Shi et al., 2021a).

**Figure 3:**
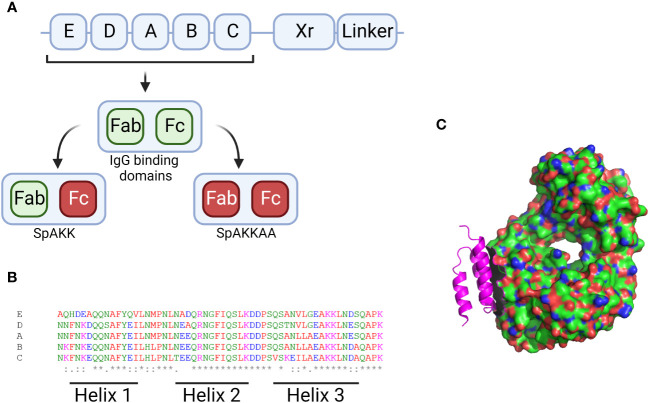
Figures displaying sequence information for SpA and its 3D structure. **(A)** Graphical description of the SpA genome with the 5 binding regions displayed by their letter. The Xr and linker regions are also shown. The Xr region is a region of variable length while the linker region contains the motifs required for binding the bacterial cell wall. Additionally the IgG binding regions for each domain are visualised along with the mutants generated when these sites are knocked out. **(B)** Aligned sequences of the 5 binding regions, the amino acids are marked up by colour and alignment is displayed along the bottom with * denoting perfect alignment and: denoting close alignment. The regions related to each helix are noted along the bottom. **(C)** 3D structure of the SpA protein (in pink) in complex with the Fab fragment of an IgM molecule. Produced in Pymol using structure 1DEE (Graille et al., 2000). Created with BioRender.com.

SpA protein consists of five homologous Ig-binding domains, as shown in **Figure 3**, that together fold into a three-helix bundle (Graille et al., 2000). This folded structure is able to bind firmly to both the fragment crystallisable (Fc) and the fragment antigen-binding (Fab) region of human antibodies (Lee et al., 2021). These can be defined by their heavy-chain-variable (VH) region, with SpA having a particular affiliation for VH3 (Kumar et al., 2015). The binding region shows a lower degree of variability, however epidemiological analysis of SpA distribution typically focuses on the variable-number tandem-repeat region, shown in **Figure 3** as ‘Xr’. This is the most variable region of SpA, where multiple intragenic recombination, non-synonymous mutation and duplication events have occurred, all of which could lead to increased pathogen fitness (Santos-Junior et al., 2016). SpA typing is occasionally unable to characterise isolates of *S. aureus* with mutations within the IgG binding domain, as this overlaps with the binding site of the primer (Baum et al., 2009). This same study also found that a number of mutants could not be typed due to the total absence of SpA. Recently it was shown that these SpA^-^ phenotypes can be due to a mutation in the 5’ untranslated region of *spa* in the ribosome binding site (Brignoli et al., 2019). The absence of SpA leads to an increase in capsule production and, critically, an increase in phagocytic uptake and subsequent host killing of the bacteria. Alternative primers have been developed to allow for the typing of Xr mutants, which, when deployed in clinical settings have identified previously non-*spa* typable isolates. This raises the potential for under-representation of *spa* mutants amongst historical studies characterising clinical *S. aureus* strains (Votintseva et al., 2014; Kareem et al., 2020).

Other *spa* mutations have been found to occur in the linker region (also known as Xc), producing SpA that is unable to anchor to the cell wall. Following translation, SpA is inserted into the membrane via the LPXTG motif where it can act as a cell wall protein. SpA can then be processed by murein hydrolases to be released as a secreted protein from the cell wall together with some peptidoglycans linked to its C terminus (Becker et al., 2014). In addition, recent data have shown that the cross wall deposition of SpA prior to secretion is reliant on production of lipoteichoic acid (LTA), a glycerol phosphate polymer which is a key component of the staphylococcal envelope. Reliance on the presence of LTA in turn provides spatial regulation of SpA deposition, restricting its deposition to the septum of dividing cells (Zhang et al., 2021). SpA-containing isolates with Xc mutations skip the cell wall protein stage and move directly to a secreted form; they do, however, feature an absence of C terminal peptidoglycans, which may have some effect on their ability to act as effective superantigens (Sorum et al., 2013; Shi et al., 2021b). As a major constituent of Gram-positive bacterial cell walls, and a key virulence factor in its own right, this additional close association with SpA regulation makes the role of LTA in pathogenesis potentially very important. Previous work has studied the role of hyperimmune polyclonal and monoclonal antibodies targeting LTA in the prevention of staphylococcal sepsis in low birthweight neonates where 75% of late-onset infections can be due to staphylococci, predominantly coagulase-negative staphylococci (CoNS) (Benjamin et al., 2006; Weisman, 2007). An effort to produce such a monoclonal antibody by BioSynexus (ClinicalTrials.gov/NCT00646399) moved into phase 2b/3 trial but failed to reduce mortality and was eventually abandoned (Patel and Kaufman, 2015).

### 4.2 SpA as a superantigen

SpA is best known as an extremely effective superantigen, predominantly affecting B-cells: while other antigens can bind ~0.01% of human B cells SpA is able to bind up to 30% of available B cells (Silverman et al., 1993). Further studies by Pauli and colleagues showed non-antigen specific B-cell clonal expansion skewed towards VH3 generation, suggesting that SpA can affect immunodominance (Pauli et al., 2014). This is in contrast to earlier findings which suggested ablation of B cells following VH3 expansion (Goodyear and Silverman, 2005): however other studies suggest responses in murine models may differ from humans in this regard. Shi and colleagues found that the presence of peptidoglycan was essential in the binding of B cells to SpA, which was in turn reliant on the LysM domain and LPXTG motif at the C-terminus (Shi et al., 2021a). The capacity of SpA to readily bind to B cells results in cross-linking of the membrane-associated B cell receptor (BCR) and lattice formation involving both Fab- and Fc-mediated interactions (Ulloa-Morales et al., 2018). The *in vivo* clinical implications of this process are unclear, and patients convalescing from *S. aureus* skin and soft tissue infection have no evidence of depleted circulating *S. aureus* memory B cell populations (Pelzek et al., 2018). This process could theoretically impact the ability of the host to form antibodies against other *S. aureus* proteins however, and to maintain a suitable memory B cell repertoire for effectively countering future exposure to infection (Goodyear and Silverman, 2003; Ulloa-Morales et al., 2018).

The repertoire of antibodies developed against other *S. aureus* antigens is reduced due to the superantigen properties of SpA. This interferes with both the earlier humoral response during acute infection and the development of long-term immune memory, potentially resulting in repeated and/or persistent infection. This raises the potential for development of a *S. aureus* vaccine against SpA protein with the aim of undermining its abilities as a superantigen. This concept has been demonstrated by vaccine candidates against other *S. aureus* superantigens, for example the fusion protein TBA_225_, which incorporates components of the toxins staphylococcal enterotoxin A (SEA) and B (SEB) and toxic shock syndrome toxin 1 (TSST-1) (Venkatasubramaniam et al., 2019).

To explore this a SpA mutant (known as SpA_KKAA_) was generated with amino acid substitutions of glutamine at positions 9 and 10 instead of lysine, and alanine at positions 36 and 37 instead of aspartate within the first of the five Ig-binding domains of SpA (Kim et al., 2010). Kim and colleagues showed that these changes can completely abrogate the ability of SpA_KKAA_ to bind both the Fc and the Fab regions of IgG. Immunization of mice with SpA_KKAA_ unleashed a broad antibody response to many different staphylococcal antigens and prevented B cell apoptosis (Kim et al., 2010). Furthermore, monoclonal antibodies raised against the SpA_KKAA_ mutant also showed effectiveness when used as prophylaxis to prevent infection with MRSA. This work was entirely performed in murine models however, so whether this would translate to human biology is not yet known. Further work by this group showed evidence of antibody generation against the SpA_KKAA_ mutant which resulted in protection from infection and reduced staphylococcal abscess formation (Kim et al., 2012). In addition to the evidence in mice, SpA_KKAA_ antibodies promote opsonophagocytic killing of MRSA in human blood, a key feature that is absent in patients with neutrophil deficiencies (Boero et al., 2021).

In more recent work, SpA_KKAA_ mutants have been used to discover potential alternative epitope sites on the SpA protein that could be utilised as antigens. Radke and colleagues identified antibodies against SpA_KKAA_ from the serum of both healthy individuals and those recovering from *S. aureus* infections, with SpA-specific memory B cells showing a strong VH3 preference (Radke et al., 2021). This study built on previous work outlining the importance of VH : VL genes in B cells and the preference for VH3 genes when targeting *S. aureus* (Roben et al., 1995). A single isolate, SA101, was proposed to induce antibodies against a novel site on SpA and was found to be the only isolate to have its binding to VH3 sites inhibited by SpA_KKAA._ This could indicate a novel epitope site not located within either the Fc or FAB binding portion of SpA due to the ability of SpAKKAA to inhibit VH3 binding. Although the substitutions made in SpA_KKAA_ have been shown to have little effect on the solubility or folding of the three-helix bundle, the changes did demonstrate an impairment in the ability of SpA in binding to B cells (Kim et al., 2010). This could account for the apparent alternative epitope binding of the isolate identified by Radke and colleagues (Radke et al., 2021): further crystallographic work is needed to determine the binding interactions between SpA_KKAA_ and antibody Fc/Fab regions. This work demonstrated the potential ability of the immune system to form antibodies against alternate sites, potentially providing new targets amenable to vaccine development.

Other recent work has attempted to refine the SpA_KKAA_ mutant and identified that the ability to crosslink VH3-IgG and VH3-IgE was maintained by the mutant. Two new candidates were identified that featured three instead of four amino acid changes, with the mutants shown to be detoxified and unable to crosslink VH3 antibodies (Shi et al., 2021a). There is therefore considerable scope to investigate the potential of other antibodies raised against other SpA epitopes, which should include studies to assess binding affinities and kinetics. Additional assessment of potential differences in alternative epitope antibodies between patients with acute infections and those with persistent infection could assess whether a different repertoire of antibodies is generated following recurrent infection.

X-Biotech have developed 514G3, a monoclonal antibody against SpA for the treatment of *S. aureus* bacteraemia initially isolated and cloned from a healthy human donor (Varshney et al., 2018). In order to counter the ability of SpA to bind strongly to IgG, an isotype of the only form that is poorly bound (IgG3) was proposed and the resulting mAb was found to opsonize SpA and facilitate natural immune-mediated clearance in a murine model (Varshney et al., 2018). The potential use of this approach as a therapeutic was supported by data showing that human IgG3 sera was more effective at inducing phagocytosis than IgG1 (Boero et al., 2022). 514G3 was taken forward to Phase I-II trial in hospitalised blood infection adult patients who were randomised 3:1 (514G3 vs placebo) for dose escalation and 40 mg/kg was the selected dose for the phase II portion of the study. Subjects enrolled in the phase II trial were randomised at a ratio of 2:1 with a total of 52 patients and, despite the limiting factor of a small population, the incidence rate of serious adverse events was found to be lower in the treated group, with early efficacy being signalled. The trial demonstrated the potential efficacy of 514G3 which was safe and well-tolerated at the doses studied, allowing for further trials of the antibody (Huynh et al., 2016).

### 4.3 Depletion of B cell repertoire

The effects of SpA as a superantigen involve not only the prevention of antibody formation but also the depletion of the B cell repertoire through multiple methods, including their crosslinking and activation which promotes B cell apoptosis. It was shown by Goodyear and Silverman that B cell apoptosis was induced by the crosslinking of SpA at B cell receptors containing VH sites with the Fab domain of SpA, ruling out the involvement of the Fc binding domain (Goodyear and Silverman, 2003). Of note, this work was performed in a murine model and therefore further investigation to confirm similar mechanism(s) in humans is still required (Pauli et al., 2014). Further studies showed that down-regulation of important markers such as CD21 and CD19 followed B-cell crosslinking, which was mirrored in superantigen-induced apoptosis by other superantigens targeting B cells (Goodyear et al., 2004). This was shown to be particularly prevalent in marginal zone B cells (Goodyear and Silverman, 2005).

Recent work investigating the involvement of SpA in B cell depletion has revealed the role that circulating IgG plays in supporting and potentiating the superantigen properties of SpA. This is primarily mediated through the Fab domain binding of the B cells and the subsequent binding of the same SpA protein to circulating IgG, with this pattern repeating to build up a lattice of crosslinked B cells, SpA, and IgG that leads to apoptosis within 72 hours in a murine model (Ulloa-Morales et al., 2018). The binding of circulating IgG antibody plays an additional role in the evasion of the host’s immune defences, as this prevents the formation of antibody hexamers, an important stage of the adaptive response that is specifically blocked by SpA (Cruz et al., 2021).

**Figure 4:**
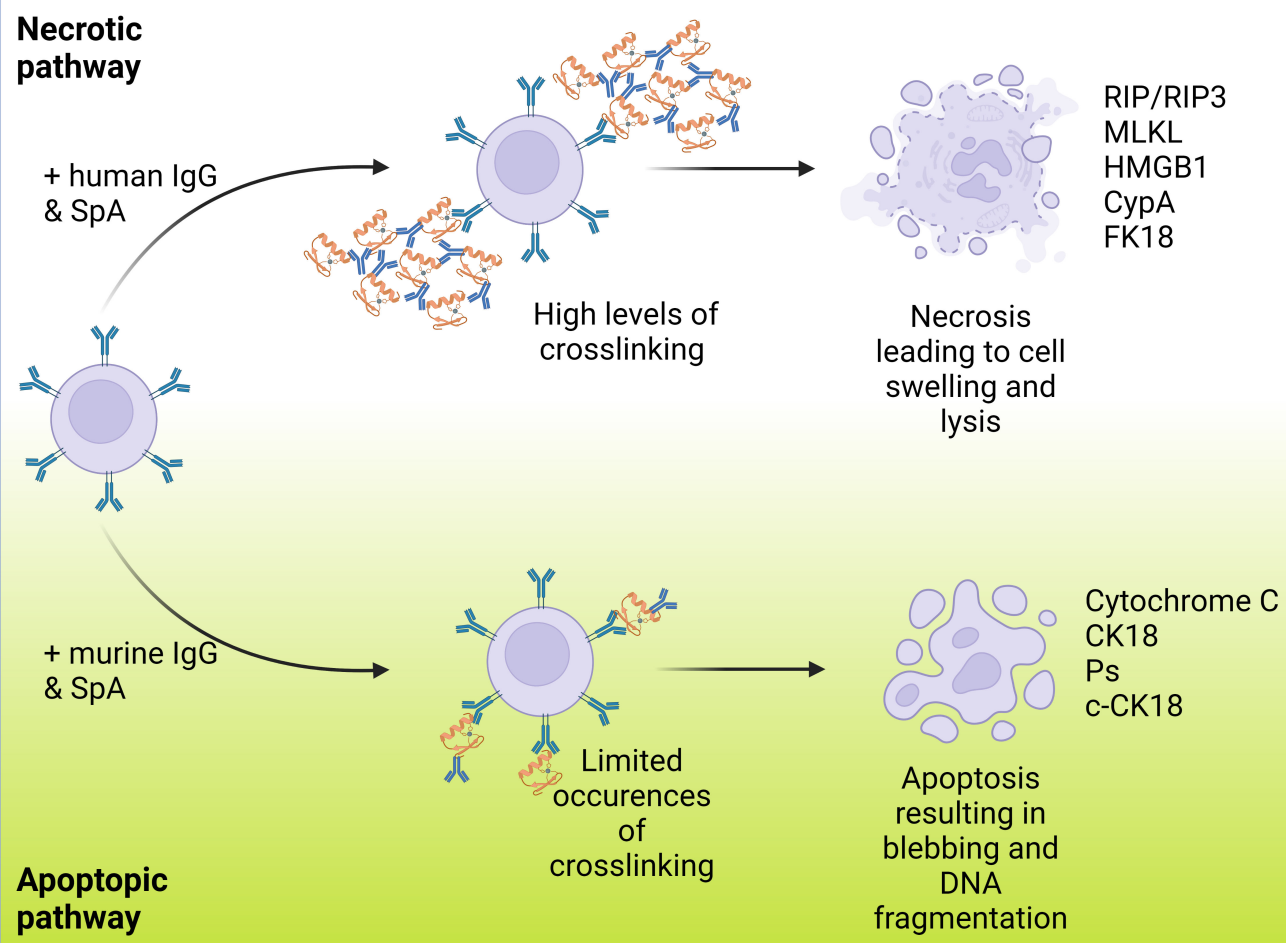
Varying routes of B cell death resulting from SpA binding and ending in either necrosis or apoptosis. This shows the paths that B cell death can occur via with B cells represented in lilac, IgG represented in blue and SpA represented in orange. B cell death can occur via either apoptosis or necrosis with each resulting in a markedly different manner of cell death displaying multiple different cell markers. Fox et al., reported that the previously noted apoptosis method was resultant of murine IgG and when instead carried out with human IgG necrosis was the primary method of cell death. A higher degree of crosslinking is noted in the necrosis case.

A new mechanism of cell death, necrosis, was recently proposed as a human immune response to SpA. Fox and colleagues found that the immune complexes formed with human IgG led to necrosis rather than apoptosis (Fox et al., 2021). It has been suggested that apoptosis occurs when in SpA forms complexes with murine IgG, while necrosis occurs when it binds to human IgG, as shown in **Figure 4**. It was also suggested that this pathway could affect not only B cells but may also trigger cell death in other leukocytes, although this has yet to be confirmed. IgG binding also has relevance to the complement system, as recent research has demonstrated strong links between the formation of IgG hexamers and initiation of the classical pathway. To initiate the antibody-led classical pathway C1q binds to the Fc region of the antibody and is joined by C1r and C1s to form the C1 complex: recent work has illustrated the importance of antibody hexamers in this process, particularly for IgG antibodies (Strasser et al., 2019). The ability of SpA to block the complement cascade in this way was confirmed by Cruz and colleagues, however they also found that IgG3 (which is not recognised by SpA) was able to engage C1q and initiate phagocytic killing of *S. aureus* (Cruz et al., 2021). This work provides some lines for investigation, particularly into the therapeutic use of IgG3, and also around the role that SpA plays in the interruption of the complement cascade compared to the other proteins previously discussed and the balance between their effects.

### 4.4 Disruption of long-lived plasma cells formation

Another aspect of the immune evasion mechanism deployed by SpA is disruption of the formation of long-lived plasma cells (LLPC). LLPCs are B cells that have migrated to the lymph node to mature and then moved into the bone marrow to secrete antibodies and provide long term immune memory. The survival of LLPCs is tightly controlled and they exist in a niche regulated by multiple factors including IL-5, TNF-ɑ, and CD44 (Cassese et al., 2003). It has been suggested that there is a relationship between SpA and the transcriptional products of the B cells they associate with, in particular those involved in the differentiation into LLPCs and the subsequent maintenance of the niche.

One factor, aiolos, a member of the Ikaros transcription factor family, has been long known to play a key role in the differentiation of B cells into LLPCs rather than to shorter-lived plasma cells (Corteís and Georgopoulos, 2004). Recent work by Keener et al. has shown that SpA disrupts the formation of the LLPCs, preventing the generation of long term immune memory. Keener and colleagues found that when a murine model was inoculated with wildtype *S. aureus* then subsequently challenged with a SpA knockout form, there was an increased splenic response towards the wildtype strain (Keener et al., 2017). This was shown to indicate an increased antigen presenting cell response with little conversion to LLPC. The number of bone marrow plasma cells was shown to be lower in the wildtype response, again implicating SpA as being partially responsible for this diminished response. The same authors also suggested a link between SpA and transcriptional control, due to the closely-related phenotypes of the wildtype *S. aureus* infection and the observation of loss of the *aiolos* gene expression (Keener et al., 2017). Other studies have, however, not found a defect in the numbers of LLPCs formed, instead suggesting that the defect in long term immunity is more related to issues surrounding epitope recognition and the range of epitopes that can be recognised (Pelzek et al., 2018).

### 4.5 Regulation of T cells

Along with the depletion of the B cell repertoire, SpA has additional effects on the adaptive immune response by interfering with the T cell response. *S. aureus* is known to be able to influence the T cell repertoire, in particular its ability to convert conventional CD(4)+ T cells into FOXP3 (+) CD25(+) T regulatory (Treg) cells, through the use of other *S. aureus* superantigens (Taylor and Llewelyn, 2010; Rabe et al., 2014; Lee et al., 2018). Treg cells promote a regulatory immune state by suppressing an active immune response, and can be triggered by a multitude of events, including the complement system via cytokines such as interleukin-2 (well-known as a Treg activator) (Chinen et al., 2016). It was also shown that SpA was able to induce Treg-associated cytokines, including IL-2 (Uebele et al., 2020); further work by the same group showed that not only was SpA responsible for inducing the cytokines needed to support Treg cells, it also acted directly to induce differentiation of CD4(+) T cells into Treg cells mediated by antigen presenting cells (Uebele et al., 2020). This influence over the T cell repertoire has significant repercussions for the immune response, firstly in suppressing the adaptive response while additionally reducing the available helper CD4+ T-cells in favour of Treg cells. This also contributes to the reduction in lasting immunity against SpA, as fewer T memory cells are maintained for long-lived protection. Currently our understanding of the impact of SpA on T cell immunology is relatively limited, and requires more research.

## 5 Conclusion

The ability of *S. aureus* to leverage a wide array of proteins in order to evade the immune system is key to the challenges faced by the host immune system in developing a protective long-term immune response against recurrent infection episodes. In parallel, the array of measures used to evade the host immune response has made the development of a safe and protective vaccine highly complex. Many of the described evasion proteins produced by *S. aureus* have homologues that are present in other bacteria and similarly interfere with the multiple aspects of the immune response described.

One protein of particular importance to *S. aureus* infection is SpA, a superantigen that has wide-ranging effects across many parts of the immune response. SpA plays a major role in subverting a protective adaptive immune response by preventing the formation of antibodies against other staphylococcal proteins. This is achieved through a variety of methods including its strong affinity for circulating antibodies, its influence on the B cell repertoire and the disruption of the formation of LLPCs. Further understanding of the role that SpA plays in interfering with a protective immune response may lead to new ways to circumvent these immune evasion strategies or yield new targets for vaccine candidates.

## Author contributions

All authors listed have made substantial direct and intellectual contribution to the work and approved it for publication.
